# Anti-inflammatory activity of monogalactosyldiacylglycerol in human articular cartilage in vitro: activation of an anti-inflammatory cyclooxygenase-2 (COX-2) pathway

**DOI:** 10.1186/ar3367

**Published:** 2011-06-17

**Authors:** Valentina Ulivi, Manuela Lenti, Chiara Gentili, Gabriele Marcolongo, Ranieri Cancedda, Fiorella Descalzi Cancedda

**Affiliations:** 1 Dipartimento di Oncologia Traslazionale, Istituto Nazionale per la Ricerca sul Cancro; Largo Rosanna Benzi 10, 16132, Genova, Italy; 2 Dipartimento di Oncologia, Biologia e Genetica, Universita' di Genova, Largo Rosanna Benzi 10, 16132, Genova, Italy; 3 Dipartimento di Scienze Chimiche, Università di Padova; Via Marzolo 1, 35131, Padova, Italy; 4 Istituto di Bioimmagini e Fisiologia Molecolare, Consiglio Nazionale delle Ricerche, Sezione di Genova; Via De Toni 5, 16132, Genova, Italy

## Abstract

**Introduction:**

The mono- and digalactosyldiacylglycerol (MGDG and DGDG) galactolipids have been purified from the thermophilic blue-green alga *Phormidium *sp. ETS-05 that colonizes the therapeutic thermal mud of Abano Terme and Montegrotto Terme, Italy. Both compounds present a marked composition in polyunsaturated fatty acids, mainly omega-3. The therapeutic thermal mud is applied mainly to osteoarthritic cartilage patients. In the present study the effect of MGDG treatment on proteins and factors expressed by human articular cartilage cells in culture and on pathways activated in inflammatory conditions was studied.

**Methods:**

Primary cultures of human articular chondrocytes were used at cell passage number 1 (P1). Cells were treated in serum-free medium with inflammatory cytokines in the presence and in the absence of MGDG. Western blot was performed on collected medium and on cell layers. At least three different experiments were performed on primary cultures. The quantitation of the MGDG effect was performed by densitometric scanning of Western blots. p38 Mitogen Activated Protein Kinase (p38) activation, Nuclear Factor-kappaB (NF-kB) activation and Prostaglandin E_2 _(PGE_2_) quantitation were performed by commercially available assays. Results are given as the mean values ± SD. All statistical analyses were performed using GraphPad software. The two-tailed Student's *t *-test was performed.

**Results:**

We report that MGDG: 1) represses the expression of interleukin-6 (IL-6) and interleukin-8 (IL-8) induced by interleukin-1alpha (IL-1α) or IL-1α + tumor necrosis factor α (TNFα) interfering with the p38 and NF-kB pathways; 2) is not toxic for the cells and does not affect the cell phenotype; 3) strongly enhances COX-2 expression induced by IL-1α or IL-1α + TNFα; 4) represses mPGES expression induced by IL-1α and the synthesis of PGE_2 _and induces the synthesis of 15-deoxy-Δ 12,14-prostaglandin J_2 _(15ΔPGJ_2_). In addition, the COX-2 product 15ΔPGJ_2 _added to the cells: 1) strongly represses IL-6 and IL-8 induced by IL-1α; 2) represses mPGES expression induced by IL-1α and the synthesis of PGE_2_.

**Conclusions:**

All together these data suggest that MGDG has an anti-inflammatory activity in human articular cartilage and possibly activates an anti-inflammatory loop triggered by COX-2 via 15ΔPGJ_2 _production, indicating a possible role of COX-2 in resolution of inflammation. The purified compound is a novel anti-inflammatory agent potentially active for human articular cartilage pathologies related to inflammation.

## Introduction

MGDG and DGDG (mono- and digalactosyldiacylglycerol galactolipids with a high content of polyunsaturated fatty acids, mainly omega-3 s) are the most widespread non-phosphorous polar lipids in the biosphere and account for 80% of the membrane lipids found in green plant tissues. In contrast with membranes of animals and yeasts where phospholipids are abundantly represented, these lipids are major constituents of the photosynthetic membranes of higher plants, algae and bacteria [1]. In animals, MGDG and DGDG are present at low concentration, particularly in the myelin and in oligodendrocytes [2]. Among specific biological activities, including anti-viral [3], anti-tumor and anti-proliferative activity [4,5], this class of compounds revealed an anti-inflammatory activity *in vitro *by inhibiting the generation of a superoxide anion in primed leucocytes, showing an inhibitory effect on the chemotaxis of human peripheral blood neutrophils *in vitro *[5,6]. An acute *in vivo *anti-inflammatory activity of this compound in a murine chronic dermatitis model was also reported [7]. MGDG and DGDG have been purified from the thermophilic blue-green alga *Phormidium *sp. ETS-05 that colonizes the therapeutic thermal mud of Abano Terme and Montegrotto Terme, Italy [8]. The therapeutic thermal mud is applied mostly to osteoarthritic cartilage patients. An *in vivo *anti-inflammatory activity of the purified compounds has been demonstrated in both the croton-oil-induced ear edema and the carrageenan-induced paw edema mouse models [9]. Indeed, some anti-inflammatory action of the thermal mud was described also in patients by monitoring the level of inflammatory cytokines in their serum [10], but studies on a possible specific anti-inflammatory action of the purified compounds at the level of the articular cartilage are missing. In a recent publication, we tested the anti-inflammatory role of MGDG in an avian model and we showed that the treatment of avian articular chondrocytes with MGDG suppressed the expression of the inflammation induced proteins Ex-FABP, Avidin, and Serum Amyloid A (SAA), in agreement with a strong anti-inflammatory property of MGDG [11].

In a previous study we described the synthesis of IL-6 and IL-8 by human articular cartilage chondrocytes following induction with IL-1α, TNFα, and the combination of the two cytokines [12]. We also reported the existence of an anti-inflammatory pathway involving COX-2 in the MC615 murine chondrocytic cell line [13]. We previously showed that in the mouse chondrocytic cell line MC615 both NF-kB and p38 dependent pathways are activated in response to an inflammatory stimulus and that p38 is involved in the NF-kB activation [14,15]. In the present study we investigated the effect of the MGDG treatment on human articular chondrocytes and we showed an inhibition of the IL-6 and IL-8 expression induced by inflammatory cytokines concomitant to an enhancement of the COX-2 expression. The possible pathways involved in inflammation and modulated by the MGDG treatment were investigated.

## Materials and methods

### Materials

IL-1α and TNFα were from PeproTech, Rocky Hill, NJ, USA. 15-deoxy-Δ^12,14^-prostaglandin J_2 _(15ΔPGJ_2_), anti-COX-2 polyclonal antiserum and anti-mPGES-1 antibodies were from Cayman Chemical (Ann Arbor, MI, USA). Anti-IL-6, anti-IL-8 and anti-actin antibodies were purchased from Santa Cruz Biotechnology (Santa Cruz, CA, USA). Anti-actin antibody was raised against a peptide mapping at the C-terminus of actin of human origin and was reactive against both actin isoforms. Antibody against type I collagen was purchased from Developmental Studies Hybridoma Bank (Iowa City, IA, USA) and antibody against type II collagen was from NeoMarkers (Fremont, CA, USA). SB203580 was purchased from Calbiochem (Darmstadt, Germany), BAY117082 was obtained from Sigma (St. Louis, MO, USA).

### MGDG preparation

MGDG was extracted from cultures of the Phormidium sp13 ETS-05 cyanobacterium isolated from the thermal mud of Abano Terme and Montegrotto Terme, Padua, Italy as described [8]. Fresh biomasses from laboratory cultures of ETS-05 were frozen and freeze-dried. A total lipidic extract was obtained by treating the dry material with a chloroform/methanol/water mixture (100:50:7 v/v). This extract was washed with water and dried under vacuum. The residue was further fractioned between n-hexane and 10% water/methanol. The n-hexane layer was discarded and the water/methanol layer was dried. Chromatographically pure MGDG was obtained from this crude polar lipidic fraction by flash chromatography in a silica gel column using a chloroform/methanol/water/acetic acid mixture (90:10:2.5:0.5 v/v) as eluent. The identity and purity of the compound was checked by NMR and ESI-MS. MGDG was dissolved in absolute ethanol and supplemented to the cell culture medium at the indicated concentrations. Ethanol concentration in cell culture medium was 1:1000.

### Cell culture and MGDG treatment

Femoral heads of adult patients after joint replacement surgery were obtained by the Ospedale S. Antonio Recco, ASL 3 Genovese, Genova, Italy with previous informed consent of the patient. According to the existing legislation, at the time the work was performed ethics approval was not required for left-over material derived from surgery.

The work was performed on 19 primary cultures derived from hip replacement surgery mostly from old patients that had a fracture of the femur or severe osteoarthritis. The range of age was 58 to 74. Only two patients were 17- and 21-years-old and had surgery following a car accident. The cartilage of old patients was thin in the areas of contact with the joint. Articular cartilage was taken from suitable less degraded areas. We did not observe striking differences in the response to inflammatory and anti-inflammatory agents of the cultured cells between old and young patients. Articular cartilage was isolated, cleared of connective tissue and subchondral bone, minced in small pieces and rinsed in phosphate-buffered saline (PBS pH 7.2). Single chondrocytes were released by repeated enzymatic digestions (1 mg/ml hyaluronidase, 400 U/ml collagenase I, 1000 U/ml collagenase II, 0.25% trypsin in PBS). Isolated cells were pooled and cultured in adhesion in F12 culture medium containing 10% FCS.

All cultures were treated at P1 passage. Cells were washed with PBS and incubated 24 hours in serum free medium with the indicated concentrations of MGDG. The medium was then replaced with fresh medium supplemented with MGDG and inflammatory cytokines (IL-1α + TNFα or IL-1α alone). Incubation was performed for 24 hours after which the cell conditioned medium was collected and analyzed.

### Western blot analysis

For protein identification, medium aliquots were loaded on a 15% SDS-PAGE. Electrophoresis was performed in reducing conditions. After electrophoresis, the gel was blotted to a BA85 nitrocellulose membrane (Schleicher and Schuell GmbH, Dassel, Germany) according to the procedure described by [16]. The blot was saturated for 16 hours with 5% non-fat cow milk in TTBS buffer (20 mM Tris HCl pH 7.5, 500 mM NaCl, 0.05% Tween 20), washed several times with TTBS and incubated for two hours at room temperature with specific antibodies raised in rabbit. After washing, the detection was performed by a conjugated HRP-anti-rabbit IgG (Amersham, Buckinghamshire, UK) for one hour at room temperature. The blot was washed with TTBS and incubated with an enhanced chemio-luminescence substrate mixture (Amersham). The blot was then exposed to an X-ray film (Amersham) in order to obtain the image.

### Molecular analysis

Total RNA was extracted from human articular cartilage using Trizol Reagent according to the manufacturer's instructions (Invitrogen, Carlsbad, CA, USA). Reverse transcription reactions were performed in a 20 μl volume with 2 μg of total RNA. A 1:10 dilution of the resulting cDNA was used as a template to quantify the relative content of mRNA by real-time PCR (ABI PRISM 7700 Sequence

Detection System) using respective primers and SYBR Green. The following primers for real-time PCR were designed using Primer Express software:

GAPDH: forward 5'-AAAGTCGGAGTCAACGGATTTG-3'

reverse 5'-TGTAAACCATGTAGTTCAGATCGATGA-3'

Type II Coll: forward 5'-CGGCTGCACAAAACACACTGC-3'

reverse 5'-CCTTCCGCCCTGCAGAT-3'

Type I Coll: forward 5'- CAGCCGCTTCACCTACAGC-3'

reverse 5'- TTTTGTATTCAATCACTGTCTTGCC-3'

### Viability test

Articular chondrocytes were seeded in standard plastic culture dishes at a concentration of 12 × 10^3^/cm^2 ^and treated with MGDG and cytokines as described. To determine cell viability the tyazolyl blue (MTT, Sigma) method was used. The culture medium was removed and replaced with 1 ml of serum-free medium supplemented with 25 μl of MTT stock solution (5 mg/ml). After four hours incubation the medium was discarded and the converted dye was solubilized with 1 ml of absolute ethanol. Dye absorbance was measured at 570 nm.

### p38 determination

p38 determination was performed with the p38 Elisa kit by Active Motif, Carlsbad, CA, USA, that allows researchers to determine activated p38, total p38 and cell number, providing a sensitive and accurate measure.

### NF-kB activation

Binding of the NF-kB p65 subunit to the NF-kB binding consensus sequence 5'- GGGACTTTCC-3' was measured with the ELISA-based Trans Am NF-kB kit (Active Motif, Carlsbad, CA, USA) using whole cell lysates prepared from chondrocyte cultures. Preparation of cell extracts was done as recommended by the manufacturer. The Trans-Am kit employs 96-well microtiter plates coated with an oligonucleotide containing the NF-kB binding consensus sequence. The active form of the p65 subunit in the whole cell lysates was detected using antibodies specific for an epitope that is accessible only when the subunit is activated and bound to its target DNA. Specificity was checked by measuring the ability of soluble wild-type or mutated oligonucleotides to inhibit binding. Results are expressed as specific binding, that is, as the absorbance values observed in the presence of the mutated oligonucleotide minus those observed in the presence of the wild-type oligonucleotide. All measurements were performed in triplicate.

### PGE_2 _quantitation

Cell cultures were treated for 24 hours with MGDG 25 μM, the medium was then removed and MGDG 25 μM + IL-1α (100 U/ml) in serum-free condition was added for 24 hours. Control cultures, that is, untreated and only MGDG or only IL-1α treated, were performed in parallel. After the 24-hour incubation the supernatants were collected, centrifuged to remove particulate matter and stored at -80°C. Aliquots were assayed for PGE_2 _content using a PGE_2_-specific competitive EIA kit-Monoclonal (Cayman Chemical, Ann Arbor, MI, USA) according to the manufacturer's instructions. Each sample was measured in triplicate in two dilutions. Statistical analysis of the data was performed.

### 15ΔPGJ_2 _quantitation

15ΔPGJ_2 _quantitation was performed using a 15ΔPGJ_2 _-specific competitive EIA kit (Enzo Life Sciences, Inc, Ann Harbor, MI, USA) according to the manufacturer's instructions. Measurements were performed on cell serum-free media obtained as described for PGE_2 _quantitation. Each sample was measured in triplicate.

### Statistic

In this study, results are given as the mean values ± SD. All statistical analyses were performed using GraphPad software. The two-tailed Student's *t*-test was performed. A value of *P *< 0.05 was considered significant.

## Results

### MGDG represses the inflammatory response induced in human articular cells by IL-1α + TNFα without impairment of cell viability

**Figure 1:**
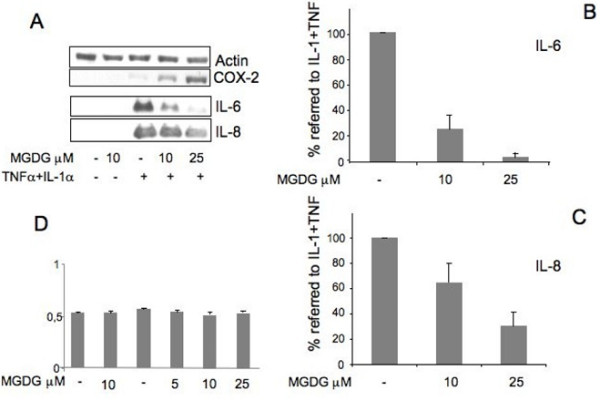
**MGDG (monogalactosyldiacylglycerol) anti-inflammatory activity on IL-1α (interleukin-1alpha) + TNFα (Tumor Necrosis Factor alpha) treated cells**. Cells were pretreated overnight with different concentrations of MGDG before treatment with 100 U/ml IL-1α + 200 U/ml TNFα for 24 hours. **A**) Upper panels: Western blot of articular cartilage cell lysates with COX-2 (Cyclooxygenase-2) antibody. Actin was blotted as an internal control. Lower panels: Western blot of cell conditioned media with IL-6 (interleukin-6) and IL-8 (interleukin-8) antibodies; **B-C**) Quantitation of the IL-6 and IL-8 repression by MGDG; densitometric analysis of five Western blots performed on five independent experiments on two chondrocyte primary cultures; to show the repression by MGDG the % value referred to the value in IL-1α +TNFα induced cells (100%) is calculated. Average ± standard deviation is shown. **D**) MTT staining of cells treated with IL-1α +TNFα in the absence and in the presence of MGDG. The experiment was performed in triplicate in two independent experiments giving similar results. One representative experiment is shown. 15ΔPGJ2, 15-deoxy-Δ^12,14^-prostaglandin J_2,_; COX-2, cyclooxygenase-2; DGDG, digalactosyldiacylglycerol; IL-1, interleukin-1; IL-6, interleukin-6; IL-8, interleukin-8; MGDG, monogalactosyldiacylglycerol; mPGES, microsomal PGE synthase; NF-kB, nuclear factor-kappaB; P1, cell passage number1; p38, p38 mitogen activated protein kinase; PGE_2_, prostaglandin E_2_; TNFα, tumor necrosis factor alpha.

In a previous study on human articular cartilage cells, we described the induction of IL-6 and IL-8 expression by treatment of the chondrocytes with IL-1α, TNFα, or the combination of the two [12]. In the present work, to study a possible anti-inflammatory activity of MGDG in cartilage, we pretreated cultures of articular cartilage cells at Passage 1 with different concentrations of the compound for 24 hours before the replacement of the medium with the medium containing IL-1α + TNFα in addition to MGDG for 24 hours. The Western blot of the cell culture media confirmed a strong induction of IL-6 and IL-8 by inflammatory cytokine treatment. Interestingly, the induction was repressed by the MGDG pre-treatment with a clear dose response mode (Figure 1A). A quantitation of the repression of the inflammatory response proteins IL-6 and IL-8 by MGDG was performed by the densitometric analysis of five Western blots performed on five independent experiments on two different chondrocyte primary cultures. To show the repression by MGDG, the % value referred to the value in IL-1α + TNFα induced cells (100%) was calculated (Figure 1B, C). The repression by MGDG was statistically significant for both proteins at the two MGDG concentrations (*P *< 0.0001). Cell viability was determined by MTT staining of the cells treated with IL-1α + TNFα in the absence and in the presence of MGDG at different concentrations. The MGDG supplement did not affect cell viability at any concentration (Figure 1D). Determination of the cell viability was performed on triplicate dishes in two independent experiments with the same result.

### MGDG represses the inflammatory response induced in human articular cells by IL-1α

**Figure 2:**
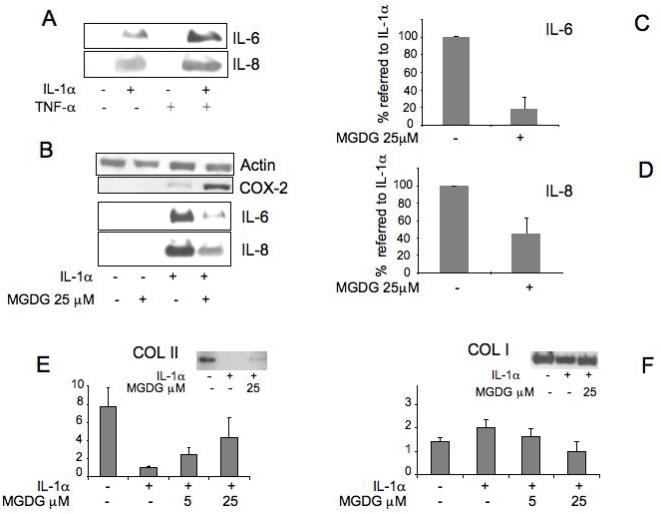
**MGDG (monogalactosyldiacylglycerol) anti-inflammatory activity on IL-1α (interleukin-1alpha) treated cells**. **A**) Human articular chondrocytes were treated with 100 U/ml IL-1α (Interleukin-1alpha) and/or 200 U/ml TNFα (Tumor Necrosis Factor alpha) for 24 hours. Western blot analysis of chondrocyte conditioned media with IL-6 (Interleukin-6), IL-8 (Interleukin-8) antibodies; **B**) Cells were pretreated 24 hours with 25 μM MGDG before treatment with IL-1α for 24 hours. Upper panels: Western blot of chondrocyte lysates with COX-2 (Cyclooxygenase-2) antibody. Actin was blotted as an internal control. Lower panels: Western blot of conditioned media with IL-6 and IL-8 antibodies; **C-D**) Quantitation of the repression of IL-6 and IL-8 by MGDG. The average of densitometric analysis of Western blots performed in six determinations on three primary cultures of human chondrocytes in duplicate dishes. To show the repression by MGDG the % value referred to the value in IL-1α induced cells (100%) is calculated. **E, F**) Real Time RT-PCR analysis of Collagen II and Collagen I mRNA expression in human articular chondrocytes treated with 100 U/ml IL-1α in the absence and in the presence of MGDG at different concentrations. Insets show Western blot analysis of chondrocyte conditioned media with Collagen type II and Collagen type I antibodies. 15ΔPGJ2, 15-deoxy-Δ^12,14^-prostaglandin J_2_; COX-2, cyclooxygenase-2; DGDG, digalactosyldiacylglycerol; IL-1, interleukin-1; IL-6, interleukin-6; IL-8, interleukin-8; MGDG, monogalactosyldiacylglycerol; mPGES, microsomal PGE synthase; NF-kB, nuclear factor-kappaB; P1, cell passage number1; p38, p38 mitogen activated protein kinase; PGE_2_, prostaglandin E_2_; TNFα, tumor necrosis factor alpha.

In order to identify the possible inflammatory pathway repressed by the MGDG treatment, adult articular cells were treated with either IL-1α or TNFα in order to distinguish the specific action of each of the two cytokines. By Western blot we showed that at the normally used concentrations IL-1α was responsible for the induction of both IL-6 and IL-8, although an increased effect was observed by the contemporary treatment with TNFα thus suggesting a synergistic action of the two cytokines (Figure 2A). MGDG repressed the induction of IL-6 and IL-8 by IL-1α (Figure 2B, C, D). The repression by MGDG was statistically significant for both proteins (*P *< 0.0001).

### MGDG sustains Collagen II expression in IL-1α treated cells

MGDG and IL-1α treated articular chondrocytes were tested for the expression of the cartilage specific Collagen II and Collagen I. A Real Time RT-PCR analysis was performed on the extracted mRNAs. The expression of Collagen II, significantly reduced by the IL-1α treatment was restored by the MGDG pre-treatment with a clear dose response mode, suggesting a protection of the cartilage phenotype as consequence of the presence of the MGDG supplement in the culture medium (Figure 2E). Type I collagen expression did not show a significant modulation by MGDG (Figure 2F). Western blot analyses are in agreement with the results obtained by Real Time RT-PCR analysis (Figure 2E, F insets)

### Repression of inflammatory pathways by MGDG in cultured chondrocytes

We previously demonstrated that in the mouse chondrocytic cell line MC615 a pathway involving p38 and NF-kB is activated in inflammation [13,15]. In order to investigate the possible signaling pathways repressed by MGDG in inflammation we treated the cartilage cells with IL-1α in the absence and in the presence of either p38 or NF-kB inhibitors.

### p38 pathway is involved in IL-6 and IL-8 production and is repressed by MGDG

**Figure 3:**
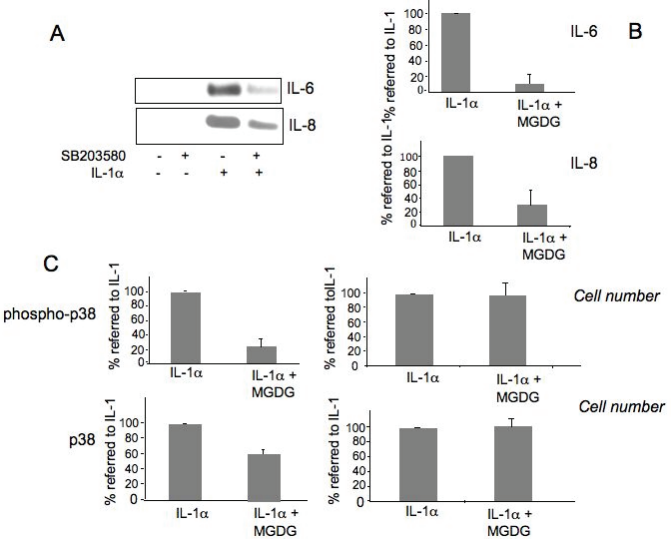
**MGDG (monogalactosyldiacylglycerol) represses p38 (p38 Mitogen Activated Protein Kinase), involved in IL-6 (Interleukin-6) and IL-8 (Interleukin-8) production**. **A**) Inhibition of IL-6 and IL-8 synthesis by the p38 inhibitor SB 203580. Human chondrocytes were treated with 10 μM SB203580 for two hours before being supplemented with 100 U/ml IL-1α for 20 hours in the presence of SB203580. Conditioned media were collected and IL-6 and IL-8 synthesis was analyzed by Western blot; **B**) Quantitation of the inhibition by the MGDG of the IL-6 and IL-8 synthesis. The average of the densitometric analysis of five western blots performed in five independent experiments on four different primary cultures is presented. To show the repression by SB203580 the % value referred to the value in IL-1α induced cells (100%) is calculated. **C**) MGDG inhibition of p38 phosphorylation. Human chondrocytes were treated with 100 U/ml IL-1α in the absence and the presence of 20 μM MGDG for 60 minutes. Phosphorylation of p38 and native inactive protein levels were assayed using the FACE p38 Kit. The average of two independent experiments performed in triplicate are shown. The number of cells in each well was determined using Crystal Violet according to the manufacturer's instructions. To show the repression by MGDG the % value referred to the value in IL-1α induced cells (100%) is calculated. 15ΔPGJ2, 15-deoxy-Δ^12,14^-prostaglandin J_2_; COX-2, cyclooxygenase-2; DGDG, digalactosyldiacylglycerol; IL-1, interleukin-1; IL-6, interleukin-6; IL-8, interleukin-8; MGDG, monogalactosyldiacylglycerol; mPGES, microsomal PGE synthase; NF-kB, nuclear factor-kappaB; P1, cell passage number1; p38, p38 mitogen activated protein kinase; PGE_2_, prostaglandin E_2_; TNFα, tumor necrosis factor alpha.

A repression of IL-6 and IL-8 was observed by treatment with SB203580 a specific inhibitor of p38 (Figure 3A, B). The repression was considered statistically significant (*P *< 0.0001).

Therefore, we wanted to measure the activation of p38 taking advantage of a commercially available ELISA assay that allows determination of phospho-p38, total p38 and cell number in the same assay. Cells were treated with IL-1α in the absence and in the presence of 20 μM MGDG. Based on the results of a preliminary time course experiment (10, 30 and 60 minutes - not shown) the assay was performed at 60 minutes. The results are shown in Figure 3C. MGDG treatment repressed the IL-1α induced increase of the phospo-p38 by 75.71% but also the total p38 protein was repressed by 36.77% while the cell number remained unchanged. The observed repression was statistically significant (*P *< 0.0001).

### NF-kB pathway is involved in IL-6 and IL-8 production and is inhibited by MGDG

**Figure 4:**
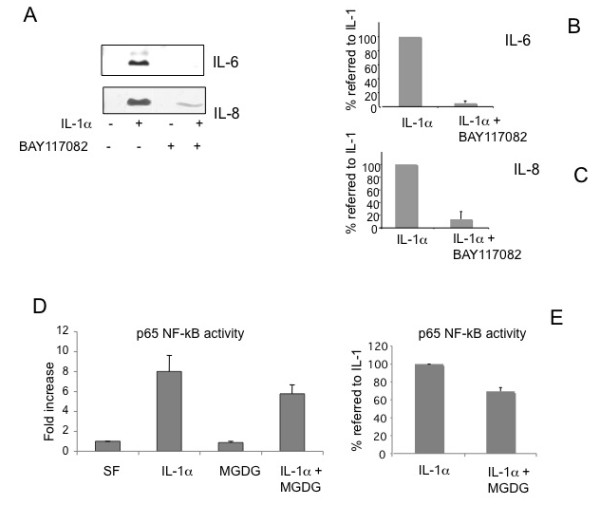
**MGDG (monogalactosyldiacylglycerol) inhibits NF-kB (Nuclear Factor-kappaB) involved in IL-6 (Interleukin-6) and IL-8 (Interleukin-8) production**. **A**) Inhibition of IL-6 and IL-8 synthesis by the NF-kB inhibitor BAY117082. Human chondrocytes were treated with 5 μM BAY117082 for two hours before being supplemented with 100 U/ml IL-1α for 20 hours in the presence of BAY117082. Conditioned media were subjected to immunoblot analysis using IL-6 and IL-8 polyclonal antibodies; **B-C**) Quantitation of the inhibition by BAY117082 of the IL-6 and IL-8 synthesis. The average of the densitometric analysis of four Western blots performed on four independent experiments on two different primary cultures is presented. To show the repression by BAY117082 the % value referred to the value in IL-1α induced cells (100%) is calculated. **D**) NF-kB activity inhibition by MGDG. Human chondrocytes were pretreated overnight with 25 μM MGDG and stimulated with 100 U/ml IL-1α in serum free conditions for 24 hours. 5 μg of whole cell lysates were tested for binding of the activated p65 NF-kB subunit to a NF-kB consensus sequence using the Trans-Am NF-kB ELISA kit. Results are expressed as specific binding. Two experiments on two different primary cultures were performed in triplicate dishes, each one assayed in triplicate. One representative experiment is shown; **E**) To show the repression by MGDG the % value referred to the value in IL-1α induced cells (100%) is calculated. Each value was subtracted of the basal value. The average of the two experiments performed in triplicate and assayed in triplicate is shown. 15ΔPGJ2, 15-deoxy-Δ^12,14^-prostaglandin J_2_; COX-2, cyclooxygenase-2; DGDG, digalactosyldiacylglycerol; IL-1, interleukin-1; IL-6, interleukin-6; IL-8, interleukin-8; MGDG, monogalactosyldiacylglycerol; mPGES, microsomal PGE synthase; NF-kB, nuclear factor-kappaB; P1, cell passage number1; p38, p38 mitogen activated protein kinase; PGE_2_, prostaglandin E_2_; TNFα, tumor necrosis factor alpha.

A repression of IL-6 and IL-8 was also observed treating the cells with BAY-117082, an inhibitor of NF-kB activation, suggesting an involvement of NF-kB in the IL-6 and IL-8 induction (Figure 4A, B, C). NF-kB activation was measured by a commercial kit based on the binding of the NF-kB p65 subunit to the NF-kB binding consensus sequence (Figure 4D, E). The experiment showed a strong induction of NF-kB activation by the IL-1α treatment and a repression of the p65 binding by MGDG of 31% ± 4.39 statistically significant (*P *< 0.0001).

### COX-2 expression is induced by treatment with IL-1α and enhanced by MGDG treatment

**Figure 5:**
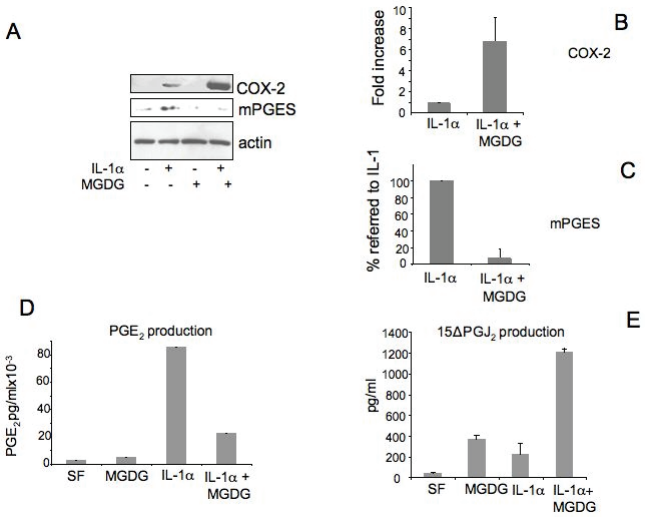
**Effect of MGDG (monogalactosyldiacylglycerol) treatment on pathways of prostaglandins production in cultured articular chondrocytes**. **A**) Human chondrocytes were pretreated overnight with 25 μM MGDG before treatment with 100 U/ml IL-1α for 24 hours. Upper panel: cell lysates were collected and analyzed by Western blot with a COX-2 polyclonal antibody. Middle panel: Western blot of cell lysates using a mPGES polyclonal antibody. Actin was blotted as an internal control; **B**) Quantitation of COX-2 expression in IL-1α + MGDG treated cells calculated as fold increase related to IL-1α only treatment. The average of three Western blots from three different primary cultures is shown; **C**) Quantitation of the inhibition of mPGES expression by the MGDG treatment. To show the repression by MGDG the % value referred to the value in IL-1α induced cells (100%) is calculated. The average of two Western blots from two different primary cultures is shown; **D**) PGE_2 _production. Conditioned media collected from human chondrocytes were analyzed for prostaglandin E_2 _content by a competitive immunoassay using the prostaglandin E_2 _EIA kit Monoclonal. Two experiments were performed in triplicate dishes, each one assayed in triplicate. One representative experiment is shown. Concentrations are expressed in pg/ml. **E**) 15ΔPGJ_2 _quantitation. 15ΔPGJ_2 _was measured in cell serum-free media using a 15ΔPGJ_2 _-specific competitive EIA Kit. Two experiments were performed, each one assayed in triplicate. One representative experiment is shown. Concentrations are expressed in pg/ml. 15ΔPGJ2, 15-deoxy-Δ^12,14^-prostaglandin J_2,_; COX-2, cyclooxygenase-2; DGDG, digalactosyldiacylglycerol; IL-1, interleukin-1; IL-6, interleukin-6; IL-8, interleukin-8; MGDG, monogalactosyldiacylglycerol; mPGES, microsomal PGE synthase; NF-kB, nuclear factor-kappaB; P1, cell passage number1; p38, p38 mitogen activated protein kinase; PGE_2_, prostaglandin E_2_; TNFα, tumor necrosis factor alpha.

Since COX-2 is part of the inflammatory response in cartilage [16], we monitored the expression of COX-2 in our experimental conditions. In all performed experiments, we observed that in cartilage cells COX-2 was induced by the treatment with IL-1α + TNFα or with IL-1α only and that its expression was strongly enhanced when cells were supplemented with MGDG before the inflammatory cytokine (CKs) addition. On the contrary, in the same experiments the expression of IL-6 and IL-8 was always strongly repressed (Figure 1, 2). Densitometric scanning of three Western blots performed on three different experiments revealed a 8.26 ± 1.23-fold increase (statistically significant; *P *= 0.0005) of the COX-2 expressed by cells treated with MGDG 25 μM + CKs over the COX-2 expressed by cells treated with CKs and no supplement of MGDG. In agreement with these data, also the COX-2 expression by cells treated with MGDG 25 μM + IL-1α compared to the COX-2 expressed by cells treated with only IL-1α showed an increase of 6.84 ± 2.19 (statistically significant; *P *= 0.0099) Figure 5B.

### mPGES expression and PGE_2 _production is induced by IL-1α and inhibited by MGDG treatment

Since mPGES is induced in cartilage in pathological and inflammatory conditions [13,17] we probed the membranes with antibodies against the protein. mPGES was expressed in cartilage cells treated with IL-1α and was repressed in the MGDG pre-treated cells (Figure 5A, C). In agreement with this finding PGE_2 _production was strongly induced by the IL-1α treatment and significantly repressed by the MGDG pre-treatment (Figure 5D). Two experiments were performed in triplicate dishes, each one assayed in triplicate. The first experiment showed a calculated repression of 49% ± 10.25 after subtraction of the basal level. The second experiment showed a calculated repression of 83.6% ± 11.26 after subtraction of the basal level. In both experiments the repression was considered statistically significant (*P *< 0.0001).

It should be noted that in the same cells where mPGES expression was inhibited, COX-2 expression was strongly induced (Figure 5A, B), suggesting that a different pathway of prostaglandin other than the PGE_2 _production was functioning in these cells, possibly leading to 15ΔPGJ_2 _production.

### 15ΔPGJ_2 _production is induced by MGDG treatment in IL-1α treated cells

There is increasing evidence of COX-2 involvement in the resolution of inflammation via its product 15ΔPGJ_2 _[18,19].

Since in the same cells where mPGES expression and PGE_2 _production was inhibited COX-2 expression was strongly induced (Figure 5 A, B), we investigated the effect of MGDG on 15ΔPGJ_2 _production. As shown in Figure 5E 15ΔPGJ_2 _production was moderately induced by either MGDG or IL-1α but was strongly increased by MGDG in the presence of IL-1α.

### 15ΔPGJ_2 _represses IL-6, IL-8 and mPGES expression induced in inflammatory conditions

**Figure 6:**
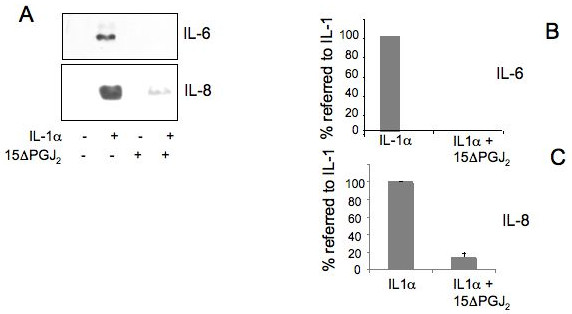
**15ΔPGJ_2 _(15-deoxy-^12,14^-prostaglandin J_2,_) represses IL-6 (interleukin-6) and IL-8 (interleukin-8) induced by IL-1α (interleukin-1 alpha)**. **A**) Human chondrocytes were treated, in the absence of serum, with 3 μM 15ΔPGJ_2 _for two hours before being incubated with 100 U/ml IL-1α for 24 hours in the presence of 15ΔPGJ_2_. A) Immunoblot analysis using IL-6 and IL-8 polyclonal antibodies were performed on conditioned media; **B**) Quantitation of the inhibition by 15ΔPGJ_2 _of the IL-6 and IL-8 synthesis was obtained by the densitometric analysis of four Western blots of four independent experiments performed in three different primary cultures. To show the repression by 15ΔPGJ_2 _the % value referred to the value in IL-1α induced cells (100%) is calculated. Average ± standard deviation is shown. 15ΔPGJ2, 15-deoxy-Δ^12,14^-prostaglandin J_2,_; COX-2, cyclooxygenase-2; DGDG, digalactosyldiacylglycerol; IL-1, interleukin-1; IL-6, interleukin-6; IL-8, interleukin-8; MGDG, monogalactosyldiacylglycerol; mPGES, microsomal PGE synthase; NF-kB, nuclear factor-kappaB; P1, cell passage number1; p38, p38 mitogen activated protein kinase; PGE_2_, prostaglandin E_2_; TNFα, tumor necrosis factor alpha.

**Figure 7:**
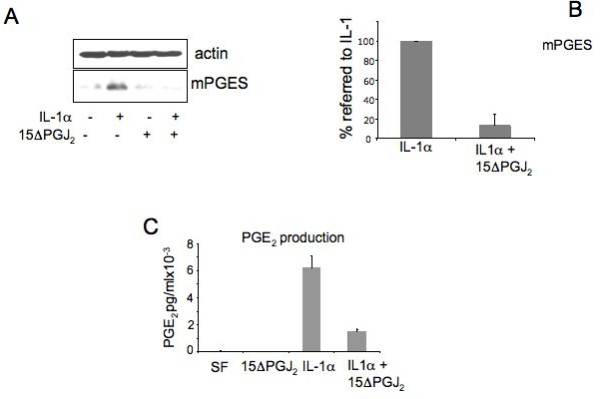
**15ΔPGJ_2 _(15-deoxy-Δ^12,14^-prostaglandin J_2,_) represses mPGES (microsomal PGE synthase) expression and PGE_2 _(prostaglandin E_2_) production**. **A**) Human chondrocytes were treated in serum free conditions with 3 μM 15ΔPGJ_2 _for two hours before being incubated with 100 U/ml IL-1α for 24 hours in the presence of 15ΔPGJ_2_. Cell lysates were subjected to immunoblot analysis using anti mPGES polyclonal antibodies. Actin was blotted as an internal control; **B**) Quantitation of mPGES inhibition by5ΔPGJ_2 _treatment. Quantitation of the inhibition by 15ΔPGJ_2 _was obtained by the densitometric analysis of three Western blots from three different primary cultures; to show the repression by 15ΔPGJ_2 _the % value referred to the value in IL-1 induced cells (100%) is calculated. Average ± standard deviation is shown. **C**) PGE_2 _production. Conditioned media collected from human chondrocytes were analyzed for prostaglandin E_2 _content by a competitive immunoassay using the prostaglandin E_2 _EIA kit Monoclonal. Two experiments were performed in triplicate dishes, each one assayed in triplicate. One representative experiment is shown. Concentrations are expressed in pg/ml. 15ΔPGJ2, 15-deoxy-Δ^12,14^-prostaglandin J_2,_; COX-2, cyclooxygenase-2; DGDG, digalactosyldiacylglycerol; IL-1, interleukin-1; IL-6, interleukin-6; IL-8, interleukin-8; MGDG, monogalactosyldiacylglycerol; mPGES, microsomal PGE synthase; NF-kB, nuclear factor-kappaB; P1, cell passage number1; p38, p38 mitogen activated protein kinase; PGE_2_, prostaglandin E_2_; TNFα, tumor necrosis factor alpha.

Therefore, we investigated the activity of 15ΔPGJ_2 _on the expression of IL-6 and IL-8 in cartilage cells treated with IL-1α. A strong decrease in the expression of IL-6 and IL-8 induced by IL-1α was observed after treatment with 15ΔPGJ_2 _(Figure 6). Densitometric scanning of three Western blots performed on three different primary cultures showed a repression statistically significant (*P *< 0.0001). In the same cells a decrease of mPGES was also observed (Figure 7A, B). Densitometric scanning of three Western blots performed on three different primary cultures showed a repression statistically significant (*P *= 0.0002). In agreement with these data also, PGE_2 _production was inhibited by 15ΔPGJ_2 _treatment (Figure 7C). Two experiments were performed in triplicate dishes, and each one assayed in triplicate. The first experiment showed a calculated repression of 53.3% after subtraction of the basal level, considered statistically significant (*P *< 0.0001). The second experiment showed a calculated repression of 76.56% after subtraction of the basal level considered statistically significant (*P *= 0.0006).

These data suggest that the 15ΔPGJ_2 _treatment drives prostaglandin synthesis toward PGD_2 _and derivatives possibly establish an anti-inflammatory loop.

## Discussion

IL-8 and IL-6 are among the key regulatory molecules of cartilage destruction in rheumatoid arthritis [20] and are present in synovial fluid of patients with osteoarthritis [21]. In a previous study on young, aged and osteoarthritic human articular cartilage, we described the induction of IL-8 and IL-6 by treatment of the cultured chondrocytes with IL-1α, TNFα, and the combination of the two [12]. It has been reported that both cytokines have a detrimental effect on articular cartilage. Indeed, IL-8 regulates leukocyte activation through p38 mitogen-activated protein kinase signaling [22], is one of the most potent chemotactic factors for neutrophils [23], and triggers neutrophil accumulation and destruction of cartilage [24]. It has also been reported that IL-8, which is up-regulated in OA cartilage chondrocytes, is involved in articular chondrocyte hypertrophic differentiation through p38 mitogen-activated protein kinase signaling causing the synthesis of an altered matrix and pathologic calcification in OA [25].

Increasing evidence suggests that IL-6 and its soluble receptor are involved in both inflammatory and degenerative joint diseases. Increased levels of IL-6 and sIL-6R have been found in synovial fluids and sera from osteoarthritis and rheumatoid arthritis patients [21] and their level correlates with the increased leukocyte infiltration in synovial tissue [26]. Furthermore, in IL-6-deficient mice immunized with type II collagen, a decrease of inflammatory cells in knee joints and a reduced antibody response to type II collagen was observed, in agreement with a crucial role played by IL-6 in the development of autoimmune collagen-induced arthritis [27]. In addition a recent study showed that IL-6 inhibited type II collagen production by rabbit articular chondrocytes through a transcriptional control, suggesting a mechanism for the phenotypic change occurring in pathological osteoarthritic cartilage [28].

We induced an inflammatory response in adult cartilage cells by a treatment with IL-1α + TNFα or IL-1α alone in the absence, and in the presence, of MGDG, and we detected and quantified the expression of IL-6 and IL-8 by these cells. We also investigated the possible inflammatory pathways repressed by MGDG. As shown by the low SD observed in the different experiments, we did not observe significant differences among the response of the cells from different patients.

The main findings of this work are: 1) MGDG represses the synthesis of IL-6 and IL-8 induced by IL-1α + TNFα in a dose response mode in cultured human articular chondrocytes; 2) the treatment with MGDG does not impair cell viability and restores type II collagen expression decreased in the inflammatory condition; 3) IL-1α is responsible for the induction of IL-6 and IL-8, although a stronger effect was observed following treatment with IL-1α + TNFα indicating a synergistic action of these two cytokines. MGDG represses also the IL-6 and IL-8 induction by IL-1α; 4) The inflammatory pathway leading to the expression of IL-6 and IL-8 following treatment with IL-1α involves the p38 and NF-kB pathways; 5) MGDG inhibits the p38 activation induced by IL-1α and partially but significantly decreases the NF-kB activation; 6) MGDG enhances the COX-2 expression induced by IL-1α + TNFα or IL-1α in cultured human articular chondrocytes.

This last point deserves attention because it is unexpected. Indeed, COX-2 is induced in inflammation and is considered one of the factors triggering an inflammatory response [29]. The fact that IL-6 and IL-8 are repressed by MGDG while COX-2 is induced suggested to us that COX-2 products could modulate the expression of IL-6 and IL-8. Because of that, we studied the expression of mPGES and measured the production of PGE_2 _and we showed *in vitro *that mPGES expression as well as PGE_2 _production were induced by IL-1α and repressed by the MGDG treatment. COX-2 is an enzyme with a well-known pro-inflammatory role as the inflammatory reaction develops concomitant to an increase of PGE_2_, but there is also evidence of its anti-inflammatory role during the resolution phase associated with the production of 15ΔPGJ_2 _[19,30,31]. In a recent paper from our laboratory an anti-inflammatory activity of 15ΔPGJ_2 _in the resolution of inflammation in the mouse chondrocytic cell line MC615 was described, showing that 15ΔPGJ_2 _was able to repress the inflammatory response to LPS possibly by PPARγ activation [13]. Measuring the concentration of 15ΔPGJ_2 _following the treatment of cartilage cells with MGDG in inflammatory condition we have detected a strong increase of 15ΔPGJ_2 _with respect to the control. Because of that, we treated cartilage cells with IL-1α in the absence and presence of 15ΔPGJ_2_, and we showed *in vitro *that this prostaglandin repressed the synthesis of IL-6 and IL-8 induced by IL-1α. In the same cells mPGES, induced by IL-1α, was repressed, suggesting the presence of an anti-inflammatory pathway that drives prostaglandin synthesis toward the synthesis of PGD_2 _and its derivatives. This hypothesis is supported by literature data showing that, in chondrocytes, 15ΔPGJ_2 _inhibited the expression of mPGES and PGE_2 _production induced by IL-1α [32]. In human mesangial cells (HMC) pre-incubation with 15ΔPGJ_2 _inhibited IL-1α-induced PGE_2 _production although IL-1α-induced COX-2 expression remained unaffected, indicating that 15ΔPGJ_2 _inhibits PGE_2 _production independently of its effect on COX-2 expression [33]. In osteoarthritic cartilage 15ΔPGJ_2 _repressed the synthesis of mPGES induced by IL-1α while COX-2 was only partially inhibited [34]. In addition, in the chronic model of collagen-induced arthritis the administration of a novel inhibitor of the mPGES expression clearly reduced PGE_2 _and mPGES expression in joint tissues, whereas COX-2 was unaffected [35]. MGDG represses mPGES, lowers PGE_2 _production, enhances COX-2 expression and induces 15ΔPGJ_2 _production; likely it activates the anti-inflammatory pathway triggered by 15ΔPGJ_2 _possibly important in inflammation resolution.

## Conclusions

**Figure 8:**
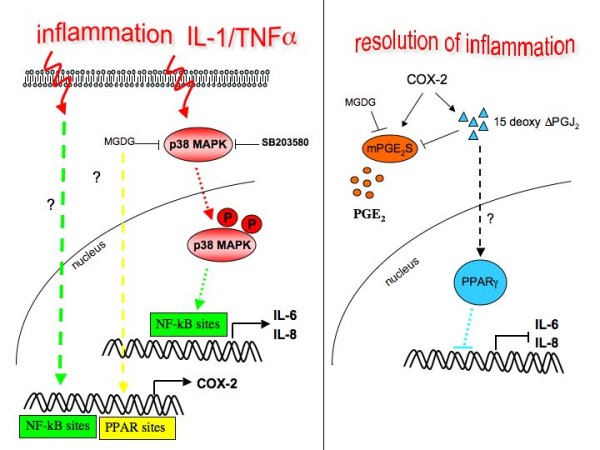
**Model for interactions possibly occurring in cultured articular chondrocytes during inflammation and resolution of inflammation**. 15ΔPGJ2, 15-deoxy-Δ^12,14^-prostaglandin J_2,_; COX-2, cyclooxygenase-2; DGDG, digalactosyldiacylglycerol; IL-1, interleukin-1; IL-6, interleukin-6; IL-8, interleukin-8; MGDG, monogalactosyldiacylglycerol; mPGES, microsomal PGE synthase; NF-kB, nuclear factor-kappaB; P1, cell passage number1; p38, p38 mitogen activated protein kinase; PGE_2_, prostaglandin E_2_; TNFα, tumor necrosis factor alpha.

In summary we here report that MGDG has a potent anti-inflammatory activity *in vitro *in cultured articular chondrocytes through the p38 and NF-KB pathways inhibition. In addition, MGDG could possibly activate an anti-inflammatory pathway involving 15ΔPGJ_2_. A cartoon rendition of the interactions proposed to occur during the inflammation and the inflammation resolution phases between the proteins and the factors considered in this manuscript is presented in Figure 8.

## Abbreviations

15ΔPGJ2: 15-deoxy-Δ^12,14^-prostaglandin J_2_; COX-2: Cyclooxygenase-2; DGDG: digalactosyldiacylglycerol; IL-1: interleukin-1; IL-6: interleukin-6; IL-8: interleukin-8; MGDG: monogalactosyldiacylglycerol; mPGES: microsomal PGE synthase; NF-kB: nuclear factor-kappaB; P1: cell passage number 1; p38: p38 mitogen activated protein kinase; PGE_2_: prostaglandin E_2_; TNFα: tumor necrosis factor alpha.

## Competing interests

This work was partially supported by a grant from the Centro Studi Termali Veneto, Pietro d'Abano di Abano Terme e Montegrotto Terme, Italy, to the University of Genova. The Centro Studi Termali holds a patent on *Phormidium *sp. ETS-05 cyanobacterium and on the active molecules produced. The salary of ML was supported by the grant from the Centro Studi Termali Veneto, Pietro d'Abano di Abano Terme e Montegrotto. All the other authors did not have any interest or support from the Centro Studi Termali Veneto, Pietro d'Abano di Abano Terme e Montegrotto.

## Authors' contributions

VU and ML started primary cultures from human cartilage samples and performed treatments, Western blots and densitometric scanning. ML performed the MTT viability test and the p38 activation assay. VU performed the NF-kB activation assay and the PGE_2 _and15ΔPGJ_2 _quantitation. Both participated in acquisition, analysis and interpretation of data and in the drafting of the manuscript. CG started primary cultures from human cartilage samples and performed Real Time RT-PCR analysis. GM purified MGDG and provided the substance. FDC and RC conceived the study, participated in its design and coordination, in acquisition and interpretation of data and helped to draft the manuscript. The decision whether to publish or not to publish the obtained results was reliant completely on FDC and RC. All authors read and approved the final manuscript.
